# Effects of light perception on visual function recovery in patients with traumatic optic neuropathy

**DOI:** 10.1038/s41598-024-54324-1

**Published:** 2024-03-29

**Authors:** Jiancun Wang, Qiang Xue, Xuewen Tan, Jie Huang, Yibai Zhu, Wen Li

**Affiliations:** 1 Department of Neurosurgery, Seventh People’s Hospital Affiliated to Shanghai University of TCM, Shanghai, China; 2 Neurosurgery Department of the First Affiliated Hospital of Soochow University, Suzhou, China; 3 Department of Neurosurgery, Eastern Hepatobiliary Surgery Hospital, Navy Medical University, Shanghai, China

**Keywords:** TON, Light perception, Visual recovery, Neuroscience, Diseases, Medical research

## Abstract

This study aimed to assess the impact of light perception presence or absence on visual function recovery in patients with traumatic optic neuropathy (TON). A retrospective analysis was conducted on the clinical data of 206 TON patients. Based on the presence or absence of light perception after injury, patients were categorized into a light perception group and a non-light perception group. A comparison was made between the two groups regarding visual acuity recovery before and after treatment. The non-light perception group comprised 63 patients, with a treatment effectiveness rate of 39.68%. The light perception group consisted of 143 patients, with a treatment effectiveness rate of 74.83%. The difference between the two groups was statistically significant (χ^2^ = 23.464, P < 0.01). Subgroup analysis indicated that surgical treatment appeared to be more effective than steroid hormone therapy for patients with light perception. Conversely, for patients without light perception, there was no significant difference in the effectiveness of the two methods. The total effectiveness rate of the light perception group was significantly higher than that of the non-light perception group, suggesting that patients with light perception before treatment experience better outcomes compared to those without light perception. Treatment choices should be individualized to ensure optimal results.

## Introduction

Traumatic optic neuropathy (TON) is a common complication in patients with cranial trauma, occurring at an incidence rate of approximately 0.5–5.0%^1,2^. The prognosis is generally poor, and without timely and effective treatment, it can result in permanent vision loss and even blindness, significantly impacting the patient's quality of life. Pathological changes associated with TON include intraneural edema of the optic nerve, microvascular occlusion or disruption of cerebrospinal fluid circulation, and interruption of direct axoplasmic transport^3,4^. Clinical manifestations often include decreased visual acuity, changes in the visual field, impaired visual evoked potentials, and a relative afferent pupillary defect, which can be readily diagnosed by experienced neurosurgeons^5^.

Currently, two primary treatment methods for TON are employed in clinical practice: high-dose steroid pulse therapy and optic canal decompression surgery. Each approach has its own advantages and disadvantages, and there is no standardized treatment protocol^6^. Optic canal decompression surgery and corticosteroids are theoretically vital for reducing intracanalicular pressure and enhancing nutrient supply to the optic nerve^7^. However, numerous issues remain unresolved in clinical practice, such as surgical efficacy, indications for surgery, and optimal timing for surgical intervention^8^. Novel treatment modalities, including stem cell therapy and neurotrophic factor therapy, are also being explored; however, most are still in the experimental stage^9,10^.

Several factors can influence the prognosis of TON, including patient age, timing of surgery, initial visual acuity after injury, and the presence of associated fractures. Among these factors, the impact of initial visual acuity after injury on the prognosis has garnered particular attention. Some studies have suggested that the severity of visual impairment following injury does not necessarily correlate positively with visual acuity recovery^11,12^. However, based on our clinical experience, we have observed significantly better treatment outcomes in patients with residual partial light perception compared to those with complete blindness. This clinical discrepancy warrants further investigation.

Therefore, in this retrospective study, we analyzed the records of 206 TON patients who underwent treatment at the Department of Neurosurgery in the Naval Medical University Affiliated Hospital and the Shanghai Seventh People's Hospital from January 2020 to December 2022. The objective of this study was to assess the impact of light perception presence or absence after injury on the recovery of visual function in patients with TON.

## Methods

### Participants

We collected clinical data from 206 patients diagnosed with traumatic optic neuropathy (TON) who received treatment at the Department of Neurosurgery in both the Navy Medical University Affiliated Hospital and the Shanghai Seventh People's Hospital between January 2020 and December 2022. The inclusion criteria were as follows: (1) diagnosis of TON; (2) underwent surgical intervention or steroid pulse therapy; (3) absence of other eye diseases or history of eye surgery; (4) stable vital signs, enabling assessment of visual acuity and other ophthalmic examinations; (5) age over 18 years. For all included cases, we recorded the initial visual acuity, gender, age, cause of injury, treatment time, and visual acuity recovery status. All methods were carried out in accordance with relevant guidelines and approved by the ethics committee of Navy Medical University Affiliated Hospital and the ethics committee of the Shanghai Seventh People's Hospital, with informed written consent for study participation obtained from all patients.

### Diagnosis

The diagnosis of TON was based on the patient's history of trauma, symptoms, and auxiliary examination results. Comprehensive ophthalmic examinations were conducted for all patients. The diagnosis was established using the following criteria: (1) traumatic brain injury, regardless of direct optic nerve injury; (2) decreased visual acuity; (3) positive relative afferent pupillary defect (RAPD), abnormal visual evoked potential (VEP), but normal fundus examination.

### Grouping

All 206 patients included in the study met the clinical diagnostic criteria for optic nerve injury. They all had a history of head and facial trauma, accompanied by varying degrees of injury to the eyelids, conjunctiva, eyeballs, and tear ducts on the affected side. Patients typically reported different levels of visual impairment, and in some cases, complete blindness. On the affected side, direct light reflex was weakened or absent, while indirect light reflex was present. On the unaffected side, direct light reflex was present, while indirect light reflex was weakened or absent. Orbital CT scans revealed orbital wall fractures in many patients, and head CT scans showed optic canal fractures in several cases. After the injury, patient vision was assessed and categorized into five levels: no light perception, simple light perception, hand motion, counting fingers, and vision > 0.05. Patients without light perception were included in the no light perception group, while those with simple light perception, hand motion, counting fingers, or vision > 0.05 were included in the light perception group.

### Management

According to post-traumatic CT results and patients' preferences, 40 patients in the no light perception group underwent optic nerve sheath decompression surgery, while 23 patients received high-dose steroid pulse therapy. In the light perception group, 91 patients underwent optic nerve sheath decompression surgery, and 52 patients received high-dose steroid pulse therapy. Classic optic nerve sheath decompression surgery techniques included transcranial optic nerve sheath decompression, intraorbital approach optic nerve sheath decompression, and endoscopic endonasal optic nerve sheath decompression. The goal of these surgeries was to alleviate optic nerve compression and achieve preservation or improvement of vision. After surgery, patients received anti-infective treatment and drugs to enhance vascular activity, nourish nerves, and other supportive therapies. High-dose steroid pulse therapy involved the administration of 500 mg of methylprednisolone within 3–8 h after the injury, followed by a 3-day continuous pulse treatment with a dose adjustment to 300 mg. This treatment was combined with dehydration, vasodilation, nutritional nerve therapy, and other approaches to promote optic nerve repair and functional recovery after injury.

### Measurements

Visual acuity was assessed before and after treatment. A visual acuity improvement of 2 grades or more after treatment was categorized as a marked effect, an improvement of 1 grade was considered effective, and no change in visual acuity was classified as ineffective. For patients with an initial visual acuity of finger counting or higher, an improvement to more than 0.2 was regarded as a marked effect. The total effective rate was calculated as (marked effect + effective) divided by the total number of cases, multiplied by 100%.

### Statistical analysis

The data were analyzed using statistical software (SPSS version 26; IBM Corp., Armonk, NY, USA). Continuous variables were presented as mean ± standard deviation (X ± S). T-tests were used to compare means between two groups for continuous variables. Categorical variables were expressed as percentages (%), and the therapeutic effective rate between the two groups was compared using the chi-square test. A P value of less than 0.05 was considered statistically significant.

### Ethical approval

The study was approved by the ethics committee of Navy Medical University Affiliated Hospital and the ethics committee of the Shanghai Seventh People's Hospital. Informed written consent for study participation was obtained from all patients.

## Results

### Baseline characteristics of patients

Table 1 provides a description of the clinical manifestations of the patients included in this study. All patients had unilateral optic nerve injuries, with 130 cases resulting from traffic accidents and 76 cases resulting from simple trauma such as falls or blows. Preoperative CT scans revealed varying degrees of orbital and cranial bone fractures in 156 patients, accounting for 75.7% of the total number of patients. Among the patients, 143 had light perception, and 63 had no light perception. In the light perception group, there were 93 males and 50 females, aged 18–69 years (mean age: 42.32 ± 12.18 years). Among them, 80 cases had injuries to the left eye, and 63 cases had injuries to the right eye. Of these, 57 cases visited the hospital within 24 h of the injury, 52 cases within 1–3 days, 28 cases within 4–7 days, and 6 cases more than 7 days after the injury. In the group without light perception, there were 36 males and 27 females, aged 21–64 years (mean age: 39.89 ± 11.01 years). Among them, 31 cases had injuries to the left eye, and 32 cases had injuries to the right eye. Of these, 37 cases visited the hospital within 24 h of the injury, 18 cases within 1–3 days, 7 cases within 4–7 days, and 1 case more than 7 days after the injury. There were no statistically significant differences in age, gender, or injured side between the two groups (P > 0.05). Attached list 1 showed each person's specific initial vision and change.

**Table 1:** Baseline characteristics of patients.

	Patients	Sex (male/female)	Age (x^- ± s)	Side(left/right)
Non-light perception	63	36/27	39.89 ± 11.01	31/32
Light perception	143	93/50	42.32 ± 12.18	80/63
χ^2^/t	–	1.164	1.363	0.799
P value	–	0.281	0.174	0.371

### Comparison of therapeutic effects between non-light perception group and light perception group

The overall effective rate of the light perception group was significantly higher than that of non-light perception group, with a statistically significant difference (χ^2^ = 23.464, P < 0.01, Table 2).

**Table 2:** Comparison of therapeutic effects between non-light perception group and light perception group.

	Patients	Marked effect (%)	Effective (%)	Ineffective (%)	Total effective rate (%)
Non-light perception	63	10 (15.87%)	15 (23.81%)	38 (60.32%)	39.68
Light perception	143	39 (27.27%)	68 (47.55%)	36 (25.17%)	74.83
	χ^2^ = 23.464, P < 0.01				

### Subgroup analysis: Comparison of treatment methods on therapeutic effects

Among the patients in light perception group, 52 received surgical treatment and 91 received steroid treatment. The overall effective rate of the surgical treatment group was higher than that of the steroid treatment group, with a statistically significant difference (χ^2^ = 8.066, P < 0.01, Table 3). Among the patients in non-light perception group, 40 received surgical treatment and 23 received steroid treatment. There was no statistically significant difference in the overall effective rate between two groups (χ^2^ = 0.363, P = 0.55, Table 4).

**Table 3:** Comparison of treatment methods on light perception group.

	Patients	Marked effect (%)	Effective (%)	Ineffective (%)	Total effective rate (%)
Surgery	52	15 (28.85%)	31 (59.62%)	6 (11.53%)	88.47
Steroid	91	24 (26.37%)	37 (40.66%)	30 (32.97%)	67.03
	χ^2^ = 8.066, P < 0.01

**Table 4:** Comparison of treatment methods on non-light perception group.

	Patients	Marked effect (%)	Effective (%)	Ineffective (%)	Total effective rate (%)
Surgery	40	7 (17.5%)	10 (25%)	23 (57.5%)	42.5
Steroid	23	3 (13.04%)	5 (21.74%)	15 (65.22%)	34.78
	χ^2^ = 0.363, P = 0.55				

## Discussion

The optic nerve, comprised of retinal ganglion cell axons, is a specialized sensory nerve with a total length of approximately 40 mm^13^. Anatomically, it can be divided into four segments: the intraocular segment (about 1 mm), intraorbital segment (about 25 mm), intracanalicular segment (5–6 mm), and intracranial segment (10 mm). External impact on any segment of the optic nerve can lead to severe nerve damage. The intracanalicular segment, which is the most delicate and represents the site where the optic nerve enters the cranial cavity, exhibits the highest incidence of injury at 71.4%^14,15^. On one hand, trauma can transmit external forces to the optic nerve canal, resulting in damage to the optic nerve due to the vulnerability of the bony structures surrounding the canal, such as the root of the lesser wing of the sphenoid and the base of the anterior clinoid process. On the other hand, trauma can cause optic nerve swelling and necrosis through compression of the optic nerve and its nutrient vessels due to bone fragment displacement or bleeding^16–18^. As a component of the central nervous system, the optic nerve possesses limited regenerative ability, and once the process of neuronal apoptosis begins, it becomes challenging to halt, leading to the death of numerous ganglion cells^19,20^.

While various eye diseases, such as optic neuritis, retinal detachment, and ischemic optic neuropathy, can result in optic nerve damage, TON is more frequently encountered by neurosurgeons, predominantly caused by car accidents, followed by falls, blows, and fights^21^. Among our group of cases, car accidents accounted for 130 cases (63.11%), falls for 32 cases (15.53%), blows and fights for 37 cases (17.96%), and other causes for 7 cases (3.4%). Optic nerve injury mechanisms in these patients can be categorized into two types: direct injury and indirect injury^22^. Direct injury often arises from violence directly impacting the outer edge of the orbit, while indirect injury frequently occurs due to optic nerve-related blood vessel spasm resulting from violence to the supraorbital margin and nasal bone^23,24^.

Following optic nerve injury, patients frequently present with clinical symptoms, such as visual field defects, impaired color vision, and potentially vision loss. In clinical practice, it is crucial to promptly identify and prioritize these symptoms, intervening early to provide treatment in order to salvage as many nerve cells as possible^25^.

Once diagnosed, immediate treatment is essential for optic nerve injuries. Currently, there is no standardized treatment protocol in clinical practice^26^. The main treatment methods include high-dose steroid pulse therapy and optic nerve decompression surgery, supplemented with diuretics, vasodilators, and drugs that improve microcirculation^27,28^. Conservative treatment often involves high-dose methylprednisolone pulse therapy. In our department, we typically administer 500 mg of methylprednisolone within 3–8 h of injury, followed by a 3-day pulse treatment with a dose adjustment to 300 mg. Steroids possess anti-inflammatory and antioxidant effects, reducing the formation of free radicals, alleviating edema reactions, and preventing vascular spasm. These properties inhibit nerve cell necrosis and protect the optic nerve^29^. Optic nerve decompression surgery involves surgically removing pressure on the optic nerve to facilitate self-repair. Surgical treatment is preferred for patients with noticeable bone fragments and hematomas compressing the optic nerve, which can include traditional transcranial optic nerve decompression surgery and intraorbital optic nerve decompression surgery^30,31^. Endoscopic optic nerve decompression surgery through the nasal cavity has also been utilized. Currently, there are no randomized controlled studies evaluating the therapeutic effects of different surgical techniques, so the primary criterion for selection remains the proficiency of the clinical physician^32^. Studies indicate that the combined effect of optic nerve decompression surgery and steroid therapy is superior to a single treatment plan, but more research is needed to substantiate this claim^33^.

In this study, there were 63 patients with no light perception after injury, yielding an effective treatment rate of 39.68%. Additionally, 143 patients had light perception after injury, with an effective treatment rate of 74.83%. The χ^2^ value was 23.464, P < 0.01, demonstrating a statistically significant difference. These findings indicate that patients who retained light perception before treatment exhibited better treatment outcomes than those who did not. Patients without light perception, regardless of steroid or surgical treatment, are unlikely to experience significant vision improvement, with low degrees of improvement observed. Existing reports suggest that patients with residual vision or light perception may have intact or partially ischemic optic nerves, with a considerable number of surviving ganglion cells^34^. Conversely, patients with no light perception may have experienced severe or irreversible optic nerve transection or necrosis, with few to no surviving ganglion cells. Hence, vision restoration is possible in the former case, emphasizing the importance of timely intervention and treatment for vision recovery. However, the possibility of vision restoration is considerably lower in the latter scenario.

The selection of treatment options for TON has long been a subject of controversy^26^. Further subgroup analysis of our study reveals that for patients with residual light perception, surgical treatment may be more effective than pure steroid therapy. Conversely, for patients without light perception after injury, the effectiveness of the two treatment options does not differ significantly. Therefore, the choice of a specific treatment plan should be individualized. For patients with residual vision after injury, regardless of visual acuity level, early and timely treatment should be administered. Steroid therapy or surgical treatment should be selected based on the actual situation to maximize ganglion cell survival and provide potential for further visual recovery. We suggest that surgery should be performed as soon as possible for clear fracture fragments, or obvious hematoma in the optic canal, or obvious indications of optic nerve compression on CT. Hormone therapy alone cannot solve the problem of optic nerve compression, and hardly help improve the patient's optic nerve function. For patients without obvious optic nerve compression, whose vision did not improve after accepting high-dose hormone treatment for 3 days, or whose vision improved first but deteriorated during hormone reduction, we still recommended early surgical treatment. However, whether or not to undergo surgery ultimately depends on the actual experience of clinicians and the wishes of patients themselves. In cases where patients have no light perception after injury, the appropriate treatment method should be carefully chosen, taking into account the potential risks of optic nerve injury or necrosis associated with surgical intervention.

## Conclusion

Prompt diagnosis and treatment are crucial for patients with TON, regardless of the chosen treatment method. This is particularly important for patients who retain light perception after the injury, as early administration of appropriate treatment can help preserve more surviving ganglion cells and promote visual recovery.

### Supplementary Information

Supplementary Information.

## Data Availability

The datasets generated for this study are available on request to the corresponding author.
