# Supplementary material for: Effects of light perception on visual function recovery in patients with traumatic optic neuropathy
Source: Sci Rep. 2024 Mar 29;14:7514. doi: 10.1038/s41598-024-54324-1 (PMC10980797; doi:10.1038/s41598-024-54324-1)
Supplement: Supplementary file 1 — Supplementary Information. [file 41598_2024_54324_MOESM1_ESM.docx]

| Number | Gender | Treatment | Initial vision | Vision after treatment |
| --- | --- | --- | --- | --- |
| 1 | F | H | 4 | 5 |
| 2 | F | S | 1 | 2 |
| 3 | M | S | 2 | 3 |
| 4 | M | S | 2 | 3 |
| 5 | M | H | 2 | 3 |
| 6 | M | H | 4 | 5 |
| 7 | F | H | 4 | 4 |
| 8 | F | H | 3 | 4 |
| 9 | F | H | 4 | 5 |
| 10 | M | S | 5 | 6 |
| 11 | M | H | 3 | 4 |
| 12 | F | S | 1 | 1 |
| 13 | M | H | 3 | 3 |
| 14 | M | S | 2 | 2 |
| 15 | M | S | 3 | 4 |
| 16 | M | H | 4 | 5 |
| 17 | M | H | 3 | 3 |
| 18 | M | H | 5 | 6 |
| 19 | F | H | 2 | 2 |
| 20 | M | S | 1 | 1 |
| 21 | F | S | 1 | 1 |
| 22 | F | S | 2 | 3 |
| 23 | M | S | 4 | 5 |
| 24 | M | S | 4 | 5 |
| 25 | M | H | 3 | 3 |
| 26 | M | H | 5 | 6 |
| 27 | M | S | 3 | 4 |
| 28 | M | S | 4 | 5 |
| 29 | M | H | 5 | 5 |
| 30 | M | S | 1 | 2 |
| 31 | F | S | 1 | 2 |
| 32 | M | H | 4 | 5 |
| 33 | M | H | 1 | 4 |
| 34 | F | S | 3 | 4 |
| 35 | M | H | 3 | 4 |
| 36 | F | H | 3 | 3 |
| 37 | F | S | 1 | 1 |
| 38 | M | S | 3 | 4 |
| 39 | M | S | 3 | 3 |
| 40 | F | H | 2 | 3 |
| 41 | F | S | 4 | 5 |
| 42 | M | H | 1 | 1 |
| 43 | F | S | 1 | 1 |
| 44 | M | H | 5 | 6 |
| 45 | M | S | 4 | 6 |
| 46 | F | S | 1 | 1 |
| 47 | M | H | 1 | 3 |
| 48 | F | H | 2 | 3 |
| 49 | M | S | 1 | 1 |
| 50 | M | S | 1 | 1 |
| 51 | F | H | 5 | 6 |
| 52 | M | H | 3 | 6 |
| 53 | M | H | 1 | 2 |
| 54 | M | H | 2 | 2 |
| 55 | F | S | 2 | 2 |
| 56 | M | S | 4 | 5 |
| 57 | M | S | 3 | 5 |
| 58 | M | H | 3 | 4 |
| 59 | M | S | 1 | 1 |
| 60 | F | H | 2 | 2 |
| 61 | M | S | 1 | 2 |
| 62 | M | H | 3 | 4 |
| 63 | M | H | 5 | 6 |
| 64 | F | S | 3 | 4 |
| 65 | M | S | 1 | 2 |
| 66 | M | S | 1 | 1 |
| 67 | F | H | 2 | 2 |
| 68 | M | H | 1 | 1 |
| 69 | F | H | 1 | 2 |
| 70 | F | S | 5 | 6 |
| 71 | F | S | 2 | 3 |
| 72 | M | S | 1 | 1 |
| 73 | M | H | 3 | 3 |
| 74 | F | H | 2 | 3 |
| 75 | M | H | 5 | 6 |
| 76 | M | H | 4 | 5 |
| 77 | M | H | 2 | 2 |
| 78 | F | H | 1 | 1 |
| 79 | M | H | 3 | 3 |
| 80 | M | S | 4 | 6 |
| 81 | M | S | 3 | 5 |
| 82 | F | H | 4 | 5 |
| 83 | M | S | 1 | 1 |
| 84 | M | S | 3 | 4 |
| 85 | M | H | 3 | 4 |
| 86 | M | H | 5 | 6 |
| 87 | M | H | 2 | 2 |
| 88 | M | H | 5 | 6 |
| 89 | M | H | 1 | 1 |
| 90 | M | S | 2 | 4 |
| 91 | F | S | 1 | 1 |
| 92 | M | S | 2 | 3 |
| 93 | F | H | 3 | 4 |
| 94 | M | H | 1 | 3 |
| 95 | F | S | 4 | 5 |
| 96 | M | H | 4 | 5 |
| 97 | M | S | 5 | 6 |
| 98 | F | S | 3 | 6 |
| 99 | M | S | 1 | 4 |
| 100 | M | S | 1 | 3 |
| 101 | M | H | 2 | 2 |
| 102 | M | S | 2 | 3 |
| 103 | M | H | 2 | 2 |
| 104 | M | S | 1 | 1 |
| 105 | M | S | 1 | 2 |
| 106 | F | S | 4 | 5 |
| 107 | M | H | 1 | 1 |
| 108 | M | H | 5 | 6 |
| 109 | F | H | 2 | 2 |
| 110 | M | H | 3 | 3 |
| 111 | M | S | 2 | 3 |
| 112 | M | S | 3 | 5 |
| 113 | F | H | 4 | 5 |
| 114 | M | H | 2 | 2 |
| 115 | M | H | 3 | 4 |
| 116 | M | H | 1 | 1 |
| 117 | M | H | 4 | 4 |
| 118 | M | H | 4 | 4 |
| 119 | M | S | 1 | 1 |
| 120 | M | H | 5 | 6 |
| 121 | M | H | 4 | 5 |
| 122 | M | H | 5 | 6 |
| 123 | F | H | 1 | 2 |
| 124 | F | S | 3 | 4 |
| 125 | F | S | 1 | 3 |
| 126 | F | S | 3 | 4 |
| 127 | F | H | 2 | 3 |
| 128 | F | S | 1 | 4 |
| 129 | M | H | 1 | 1 |
| 130 | F | S | 1 | 1 |
| 131 | M | S | 2 | 3 |
| 132 | M | S | 1 | 2 |
| 133 | M | H | 3 | 3 |
| 134 | M | H | 2 | 3 |
| 135 | M | H | 1 | 1 |
| 136 | M | H | 5 | 6 |
| 137 | M | S | 2 | 3 |
| 138 | M | S | 1 | 1 |
| 139 | F | H | 1 | 1 |
| 140 | M | S | 1 | 2 |
| 141 | F | H | 1 | 1 |
| 142 | F | H | 3 | 3 |
| 143 | F | S | 1 | 3 |
| 144 | F | S | 2 | 4 |
| 145 | M | H | 5 | 6 |
| 146 | F | S | 3 | 3 |
| 147 | F | H | 1 | 1 |
| 148 | M | S | 1 | 1 |
| 149 | M | S | 1 | 1 |
| 150 | F | S | 4 | 6 |
| 151 | F | H | 2 | 3 |
| 152 | M | S | 3 | 4 |
| 153 | F | H | 1 | 2 |
| 154 | M | H | 5 | 6 |
| 155 | F | H | 5 | 6 |
| 156 | F | H | 2 | 2 |
| 157 | M | H | 3 | 4 |
| 158 | M | H | 2 | 2 |
| 159 | M | S | 1 | 4 |
| 160 | M | H | 4 | 5 |
| 161 | M | H | 3 | 4 |
| 162 | M | H | 5 | 6 |
| 163 | M | H | 2 | 3 |
| 164 | M | S | 4 | 5 |
| 165 | M | H | 5 | 6 |
| 166 | M | H | 2 | 3 |
| 167 | F | S | 1 | 1 |
| 168 | F | S | 3 | 4 |
| 169 | F | H | 5 | 6 |
| 170 | M | S | 1 | 2 |
| 171 | F | H | 3 | 3 |
| 172 | M | H | 4 | 6 |
| 173 | M | H | 4 | 5 |
| 174 | F | S | 1 | 3 |
| 175 | M | H | 3 | 4 |
| 176 | F | S | 1 | 2 |
| 177 | M | S | 2 | 5 |
| 178 | F | H | 3 | 4 |
| 179 | M | H | 1 | 1 |
| 180 | F | S | 2 | 2 |
| 181 | F | H | 1 | 1 |
| 182 | M | H | 5 | 6 |
| 183 | F | S | 3 | 4 |
| 184 | F | H | 4 | 4 |
| 185 | F | S | 3 | 4 |
| 186 | F | S | 1 | 1 |
| 187 | F | S | 1 | 1 |
| 188 | M | H | 5 | 6 |
| 189 | F | H | 3 | 4 |
| 190 | F | S | 3 | 4 |
| 191 | F | H | 1 | 1 |
| 192 | M | H | 3 | 3 |
| 193 | M | H | 1 | 2 |
| 194 | M | S | 4 | 4 |
| 195 | M | H | 2 | 3 |
| 196 | F | S | 2 | 3 |
| 197 | F | H | 3 | 4 |
| 198 | M | H | 3 | 4 |
| 199 | M | H | 5 | 6 |
| 200 | M | H | 5 | 5 |
| 201 | F | H | 4 | 4 |
| 202 | F | S | 1 | 1 |
| 203 | M | H | 5 | 6 |
| 204 | M | S | 5 | 6 |
| 205 | F | S | 4 | 6 |
| 206 | M | H | 1 | 1 |

M:male; F:female; S:surgery; H: hormone therapy; 1:no light perception; 2:simple light perception; 3:hand motion; 4:counting fingers; 5:vision > 0.05; 6: vision > 0.2.
